# Comparative efficacy analysis of ultrasound-guided quadratus lumborum block and lumbar plexus block in hip arthroscopy: a pilot prospective randomized controlled trial

**DOI:** 10.1093/jhps/hnac020

**Published:** 2022-03-29

**Authors:** Liangjing Yuan, Chengshi Xu, Ye Zhang, Geng Wang

**Affiliations:** Department of Anesthesiology, Beijing Jishuitan Hospital, Beijing 100000, China; Department of Anesthesiology, Beijing Jishuitan Hospital, Beijing 100000, China; Department of Anesthesiology, Beijing Jishuitan Hospital, Beijing 100000, China; Department of Anesthesiology, Beijing Jishuitan Hospital, Beijing 100000, China

## Abstract

Controlled trials assessing quadratus lumborum block (QLB) for post-operative analgesia in hip surgery are scarce. This study aimed to compare ultrasound-guided QLB and lumbar plexus block (LPB) for clinical efficacy in hip arthroscopy. Patients undergoing hip arthroscopy in Beijing Jishuitan Hospital in January–June 2019 were randomized to the lumbar plexus (L) and quadratus lumborum (Q) groups (*n* = 25/group). After either ultrasound-guided block for 30 min, both groups were prepared for surgery after muscle strength measurement in the affected limbs. Opioid doses for patient-controlled analgesia (PCA), visual analog scale (VAS) scores in the resting and active states, upon leaving the post-anesthesia care unit (PACU), and at 2–48 h post-surgery were recorded, and post-operative complications were also recorded. Muscle strength in the affected limbs was significantly higher in the Q group compared with the L group (4.0 versus 2.0, *P* < 0.001). VAS scores were similar in both groups post-surgery (*P* > 0.05). One patient had epidural spread in the L group, with no other complications. Compared with ultrasound-guided LPB, ultrasound-guided QLB provides similar and good post-operative analgesia after hip arthroscopy, with less impact on muscle strength and fewer complications. These results should be confirmed in larger trials.

## INTRODUCTION

Arthroscopy has become the gold standard for the early diagnosis and treatment of hip joint diseases [1]. Indeed, hip arthroscopy is an increasingly common procedure thanks to advanced surgical tools and method refinement [2], which helps address pathology in and around the hip joint. Compared with open hip surgery, hip arthroscopy has the advantages of a small surgical incision and reduced damage to the joint cavity and surrounding soft tissues [3]. Hip arthroscopy is a comprehensive therapeutic process, and post-operative rehabilitation is crucial for its therapeutic effect; however, obvious post-operative pain limits its application [4].

Lumbar plexus block (LPB) affects the main branches of hip joint capsule nerves, including the femoral, lateral femoral cutaneous and obturator nerves, and was first introduced in 1973 [5]. LPB is an analgesic regimen after surgery involving the hip’s anterior capsule, reducing post-operative pain and opioid dose [6]. On the other hand, quadratus lumborum block (QLB), a variant of transversus abdominis plane (TAP) block, was proposed in 2007 [7]. Currently, four QLB types are available, i.e. QLB1-4 as lateral, posterior, anterior/transmuscular and intramuscular types, respectively [8]. Because L1 and L2 also run between the thoracolumbar and intra-abdominal fasciae before leaving the intervertebral foramen, injection at this site can partially block the lateral femoral cutaneous, femoral and obturator nerves branched from L2 [9]. Consequently, as a trunk block, QLB can be used for multimodal analgesia after hip surgery.

Currently, two case reports [10, 11] and some recent studies [12–14] have described QLB for hip surgery with good post-operative analgesia. Trials are also currently ongoing (e.g. ClinicalTrials.gov NCT03408483 and [15]). In addition, a recent randomized, double-blind, placebo-controlled trial demonstrated that ultrasound-guided QL3 block represents an effective pain management tool following Total Hip Arthroplasty [13]. However, the latter clinical controlled trial was a single-center study.

The hypothesis of this pilot study was that QLB is similar to LPB and can provide good post-operative analgesia for hip arthroscopy. Therefore, this pilot study aimed to assess comparatively the clinical efficacies of ultrasound-guided QLB and LPB in hip arthroscopy. The results could help find a safer and more convenient method for perioperative analgesia for hip arthroscopy.

## MATERIALS AND METHODS

### Patients

Patients admitted to the XXX Hospital for hip arthroscopy due to glenoid ligament injury in January–June 2019 were enrolled. Inclusion criteria were: planned unilateral hip arthroscopy; grade I–III American Society of Anesthesiologists (ASA) classification of physical condition [16]; 18–60 years old and body mass index (BMI) <35 kg/m^2^. Exclusion criteria were: infection at the puncture site; anatomical variation; use of anticoagulants; uncooperative position; abnormal nerve function of the affected limb; declining surgery or continuous post-operative patient-controlled analgesia (PCA).

This study was approved by the Institutional Review Board of XXX Hospital (number 201805-19) and registered in the Clinical Trial Registry (clinical trials.gov identifier: ChiCTR1900020612). Written informed consent was provided by each participant before enrollment.

### Study design

According to Randomizer Study Version 4.0 (http://www.randomizer.org/), the patients were randomly allocated in a 1:1 ratio to the lumbar plexus (L) and quadratus lumborum (Q) groups using a random number table. The random numbers were placed in an opaque envelope, which was opened by the anesthesiologist before surgery. The anesthesiologists who performed the blocks were not the same as those who monitored the patients. The anesthesiologists did not participate in patient evaluation. The surgeon and the patients didn’t know about grouping.

### Surgical procedures

**Fig. 1.**
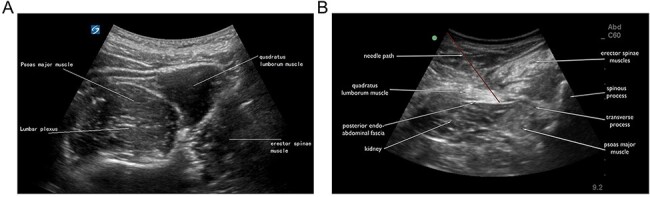
Ultrasound-guided approaches by the ‘shamrock’ method. A. Lumbar plexus block (LPB). B. Quadratus lumborum block (QLB).

The patients underwent routine fasting for 6 h before surgery. The venous access was opened in the anesthesia-preparation room, and continued oxygen inhalation with a mask (3 l/min) was conducted. Electrocardiographic features, heart rate (HR), noninvasive blood pressure (NiBP) and pulse oxygen saturation (SpO2) were monitored. Then, intravenous anesthesia with fentanyl (1–1.5 μg/kg) and midazolam (0.03–0.04 mg/kg) was performed by an anesthesiologist with >3 years of experience in ultrasound-guided peripheral nerve block. An M-Turbo ultrasound system (SonoSite, USA) in the neuroimaging mode and transducers of the L group were used to connect the C60x/5-2 MHz convex array probe to perform the ‘shamrock’ method of LPB as previously described [17]. Briefly, after the abdominal muscles were identified, the probe was moved slowly to the back and tilted to the caudal side. Typical shamrock-shaped images composed of the L4 transverse process, psoas major, erector spinae and quadratus lumborum were obtained. At this time, the hyperechoic structure in the 1/4 quadrant of the posterior psoas major was the lumbar plexus nerve (Fig. 1). The probe was fixed, and the area 4 cm away from the spinous process of the L4 was located as the puncture point. A 22G, 120-mm nerve stimulation needle tip Stimuplex D Plus (B. Braun, Germany), was guided to the lumbar plexus area and a local anesthetic was injected.

**Fig. 2.**
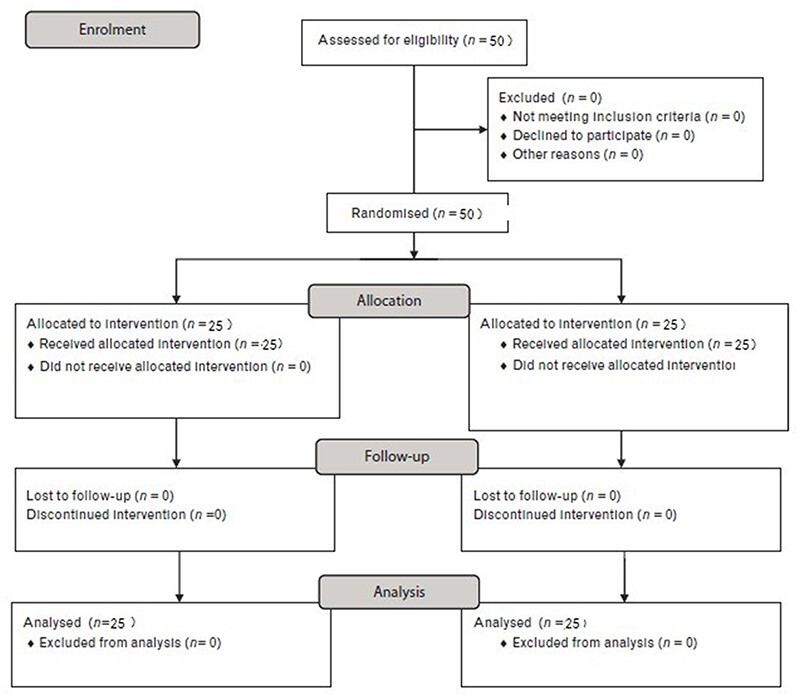
Study flow diagram. All enrolled patients completed the study.

Transducers in the Q group were connected to the C60x/5-2 MHz convex array probe, and the QLB method [7] described by Blanco was performed. In the lateral position, a low-frequency convex array probe was placed vertically above the iliac crest. Then, a 22G, 120-mm nerve stimulation needle tip Stimuplex D Plus (B. Braun, Germany) was guided to pass through the quadratus lumborum via the anteromedial direction from the back of the probe until the needle tip was located between the quadratus lumborum and the psoas major, and a local anesthetic was injected into the fascia (Fig. 2).

In both groups, the local anesthetic was 0.4% ropivacaine at 0.4 ml/kg. The blocking procedure time was recorded from the beginning of ultrasonic scanning to the end of local anesthetic injection. The block was performed for 30 min, and muscle strength of the quadriceps femoris in the affected limbs was measured. Two senior anesthesiologists performed all blocks.

Subsequently, general anesthesia was induced by target control infusion (TCI) of propofol (4.0–4.5 μg/ml), sufentanil (0.2 μg/kg) and cisatracurium (0.2 mg/kg). Then, a laryngeal mask was placed for mechanical ventilation, and TCI propofol maintained a bispectral index (BIS) of 40–55. Then, surgery was performed, and propofol infusion was discontinued immediately post-surgically. Both groups were next transferred to the PACU and underwent PCA immediately. The electronic analgesia pump (Auto Med 2000, ROK) comprised 2 μg/kg sufentanil, 10 mg tropisetron and 100 ml normal saline; the background and single doses were 2 ml/h and 0.5 ml, respectively. The lock time was 15 min.

All arthroscopies were performed by the same senior arthroscopic surgeon.

### Outcome measures

Baseline patient data were recorded, including gender, age, BMI, ASA class, complications, surgery time, block procedure time and muscle strength after block. The main outcome was the total dose of sufentanil for PCA 24 h post-surgery. Secondary outcomes were a total dose of sufentanil for PCA upon leaving the PACU, and at 2, 4, 8, 12 and 48 h post-surgery, respectively; VAS scores in the active (flexion, intorsion or extorsion) state at 4 and 24 h post-surgery, respectively; VAS scores in the resting state upon leaving the PACU, and at 2, 4, 8, 24 and 48 h post-surgery, respectively [7]; HR, SpO2, NiBP and complications within 48 h post-surgery such as epidural spread, bilateral block, post-operative nausea and vomiting, respiratory depression, itching, urinary retention, kidney damage, bleeding and hematoma at the puncture site.

The quadriceps muscle strength was recorded after 30 min of the block by the anesthesiologist, who did not perform the block but administered the anesthesia. The quadriceps muscle strength was evaluated by freehand muscle strength examination. The patient was told to take the lateral decubitus position with the affected limb on top. If the affected limb could not be kept straight after hip flexion and knee bending, the muscle strength was grade 0 (without muscle contraction) or grade 1. If the affected limb could be straightened, muscle strength was grade 2. Then the patient took the supine position with hip flexion. If the patient could straighten himself after knee bending, muscle strength was grade 3. If the ankle joint of the affected limb gave a certain resistance, muscle strength was grade 4 (mild resistance) or grade 5 (maximum resistance).

The VAS was marked according to the pain degree, between 0 (no pain) and 10 (severe pain). The VAS was conducted by a PCA group member.

### Definitions and follow up

Data collection lasted 48 h, and the patients and researchers involved in post-operative data collection were blinded to the specific anesthesia received by the patients.

### Sample size

Sample size calculation was performed with Power and sample size calculation version 3.1.2 (Vanderbilt University, Nashville, TN, USA), based on VAS scores after QLB [18] and LPB [19] for hip arthroscopy. We assumed VAS scores after QLB and LPB are similar and LPB groups, significance levels of  *α* = 0.05 and  *β* = 0.10, the sample size for each group was estimated as 22 cases. Considering a potential dropout rate of 10%, about 25 cases were needed for each group.

### Statistical analysis

SPSS version 21.0 (SPSS, USA) was used for statistical analysis. Normally distributed measurements are mean ± standard deviation (SD) and were compared by independent samples *t*-test. For repeated measurement data, repeated measures analysis of variance (ANOVA) was used to compare within groups at different time points; meanwhile, multivariate analysis of variance was used for between-group comparisons. Measurement data with skewed distribution were presented as median (interquartile range, IQR), and the rank-sum test was performed for between-group comparisons. Count data were compared by the chi-square test and ranked data by the rank-sum test. *P* < 0.05 was considered statistically significant.

## RESULTS

### Patient general information

Fifty patients were included in this trial, with 25 cases in each group. All patients completed the study. The mean patient ages in the L and Q groups were 41.4 ± 11.3 and 37.5 ± 9.5 years, respectively. Demographic characteristics, block procedure time, surgery time and other baseline features were similar in both groups (Table I).

**Table I.** General patient data

	*L group (n = 25)*	*Q group (n = 25)*	*P value*
Age (y)	41.4 ± 11.3	37.5 ± 9.5	0.183
Gender (Male/Female)	13/12	12/13	0.777
BMI (kg/m^2^)	23.9 ± 3.0	22.9 ± 2.8	0.306
ASA classification I (*n*)	20	19	0.735
ASA classification II (*n*)	5	6	0.735
Hypertension (*n*)	2	4	0.389
Diabetes (*n*)	4	3	0.687
Surgery time (min)	54.6 ± 14.9	56.8 ± 20.1	0.306
Blocking procedure time (min)	4.0 ± 2.2	4.4 ± 2.2	0.885
Muscle strength after block	2.0 (1.0–3.0)	4.0 (4.0–4.0)	*P* < 0.001

BMI: Body mass index; ASA: American Society of Anesthesiologists; L group: lumbar plexus group; Q group: quadratus lumborum group.

### Muscle strength after block

After 30 min of the block, quadriceps muscle strength was significantly increased in the Q group [2.0 (1.0–3.0)] compared with the L group [4.0 (4.0–4.0)] (*P* < 0.001; Table I).

### Opioid dose

Upon leaving the PACU, and at 2–48 h after surgery, opioid doses for PCA in the L group were 1.1 (1.9–1.6), 4.4 (4.1–4.6), 8.6 (8.1–9.1), 16.6 (16.1–17.1), 24.6 (24.1–25.1), 48.6 (48.2–49.1) and 96.6 (96.1–97.1) ml, respectively, versus 1.1 (1.0–1.3), 4.4 (4.1–6.6), 8.6 (8.2–8.7), 16.5 (16.1–16.8), 24.6 (24.1–24.7), 48.6 (48.1–48.9) and 96.6 (96.2–96.7) ml for the Q group, respectively, indicating no significant differences (Table II).

**Table II.** Cumulative doses of PCA at different time points after surgery (ml)

	*L group (n = 25)*	*Q group (n = 25)*	*P value*
Leaving the PACU	1.2 (1.0–1.6)	1.1 (1.0–1.3)	0.372
2 h after surgery	4.4 (4.1–4.6)	4.4 (4.1–4.6)	0.674
4 h after surgery	8.6 (8.1–9.1)	8.6 (8.2–8.7)	0.761
8 h after surgery	16.6 (16.1–17.1)	16.5 (16.1–16.8)	0.430
12 h after surgery	24.6 (24.1–25.1)	24.6 (24.1–24.7)	0.538
24 h after surgery	48.6 (48.2–49.1)	48.6 (48.1–48.9)	0.442
48 h after surgery	96.6 (96.1–97.1)	96.6 (96.2–96.7)	0.552

PCA: Patient-controlled anesthesia; L group: lumbar plexus group; Q group: quadratus lumborum group.

### QLB and LPB cause similar levels of pain in hip arthroscopy

Resting VAS scores in the Q group were 4.0 (3.0–6.0) immediately after surgery, and 3.0 (2.0–4.0), 2.0 (1.5–3.0), 2.0 (0.5–2.5), 0 (0–1.0) and 0 (0–0) at 2–48 h after surgery, respectively, with no significant differences compared with the L group at various time point after hip arthroscopy [4.0 (0.5–5.0), 2.0 (1.0–4.0), 2.0 (0–3.5), 1.0 (0–2.5), 0 (0–0.5) and 0 (0–0), respectively; (Table III)].

**Table III.** VAS scores at different time points after surgery

		*L group (n = 25)*	*Q group (n = 25)*	*P value*
Resting state VAS	Leaving the PACU	4.0 (0.5–5.0)	4.0 (3.0–6.0)	0.066
	2 h after surgery	2.0 (1.0–4.0)	3.0 (2.0–4.0)	0.07
	4 h after surgery	2.0 (0–3.5)	2.0 (1.5–3.0)	0.081
	8 h after surgery	1.0 (0–2.5)	2.0 (0.5–2.5)	0.233
	24 h after surgery	0 (0–0.5)	0 (0–1.0)	0.347
	48 h after surgery	0 (0–0)	0 (0–0)	0.615
Active state VAS	4 h after surgery			
	Flexion	3.0 (1.0–5.0)	3.0 (3.0–4.0)	0.322
	Intorsion	2.0 (0.5–4.0)	2.0 (2.0–4.0)	0.106
	Extorsion	2.0 (0.5–4.5)	2.0 (1.5–3.5)	0.478
	24 h after surgery			
	Flexion	1.0 (0.5–2.5)	1.0 (1.0–2.0)	>0.999
	Intorsion	1.0 (0–2.0)	1.0 (0–1.0)	0.496
	Extorsion	1.0 (0–2.0)	0 (0–1.0)	0.388

VAS: Visual analog scale; PACU: post-anesthesia care unit; L group: lumbar plexus group; Q group: quadratus lumborum group.

Flexion, intorsion and extorsion VAS scores (0 to 10 points) at 4 h after surgery in the Q group were 3.0 (3.0–4.0), 2.0 (2.0–4.0) and 2.0 (1.5–3.5), respectively, with no significant differences compared with the L group [3.0 (1.0–5.0), 2.0 (0.5–4.0) and 2.0 (0.5–4.5), respectively; (Table III)]. Flexion, intorsion and extorsion VAS scores (0 to 10 points) at 24 h after surgery in the Q group were 1.0 (1.0–2.0), 1.0 (0–1.0) and 0 (0–1.0), respectively, with no significant differences compared with the L group [1.0 (0.5–2.5), 1.0 (0–2.0) and 1.0 (0–2.0), respectively); (Table III)].

### Secondary outcomes

NiBP, HR and SpO2 during surgery were similar in both groups (Table IV). For post-operative complications, one patient had epidural spread (4%) in the L group; there were no other complications such as nausea and vomiting in the whole study population (Table V).

**Table IV.** Noninvasive blood pressure, heart rate and blood oxygen saturation at different points during surgery

		*L group (n = 25)*	*Q group (n = 25)*	*P value*
Systolic pressure	Entering the room	129.0 ± 7.6	127.2 ± 7.2	0.852
	Laryngeal mask insertion	113.0 ± 9.7	110.6 ± 9.4	0.915
	Starting the surgery	106.9 ± 7.6	105.3 ± 6.7	0.539
	Completing the surgery	105.7 ± 8.7	104.5 ± 6.6	0.232
Diastolic pressure	Entering the room	73.7 ± 9.3	72.44 ± 9.4	0.91
	Laryngeal mask insertion	64.4 ± 8.1	63.0 ± 8.4	0.806
	Starting the surgery	60.0 ± 6.6	59.5 ± 6.9	0.64
	Completing the surgery	58.8 ± 8.7	60.2 ± 7.0	0.511
Heart rate	Entering the room	79.8 ± 5.6	78.6 ± 6.3	0.284
	Laryngeal mask insertion	65.5 ± 4.7	66.0 ± 6.3	0.06
	Starting the surgery	60.8 ± 4.8	63.6 ± 6.2	0.177
	Completing the surgery	60.2 ± 6.0	63.3 ± 6.7	0.297
Blood oxygen saturation	Entering the room	99.0 ± 0.8	98.8 ± 0.9	0.329
	Laryngeal mask insertion	100.0 ± 0.0	100.0 ± 0.0	–
	Starting the surgery	100.0 ± 0.0	100.0 ± 0.0	–
	Completing the surgery	100.0 ± 0.0	100.0 ± 0.0	–

L group: lumbar plexus group; Q group: quadratus lumborum group.

**Table V.** Post-operative complications

	*Q group (n = 25)*	*L group (n = 25)*	*P value*
Epidural spread	0	1	>0.999
Bilateral block	0	0	–
Post-operative nausea and vomiting	0	0	–
Respiratory depression	0	0	–
Itching	0	0	–
Urinary retention	0	0	–
Kidney damage	0	0	–
Bleeding and hematoma at the puncture site	0	0	–

L group: lumbar plexus group; Q group: quadratus lumborum group.

## DISCUSSION

This study demonstrated that patients undergoing QLB had significantly higher muscle strength in the affected limbs compared with those administered LPB, with no significant differences in sufentanil dose at various time points after surgery, as well as VAS scores in the resting and active states.

Although hip arthroscopy is a minimally invasive procedure, like other open surgeries, post-operative pain remains an important issue [20]. Therefore, it was proposed that an effective analgesia method must include the basic contents for post-operative rehabilitation in hip arthroscopy, which can directly affect the surgical effects and the long-term function of the joint [21].

Previous studies have shown that LPB can reduce post-operative pain and opioid dose after total hip arthroplasty [22]. For example, a randomized trial of patients undergoing hip fracture repair showed that LPB reduces pain scores and improves patient satisfaction [23]. In addition, ultrasound-guided LPB by the ‘shamrock’ method [17] could effectively avoid the interference of bony structure acoustic shadow. Studies by Gürkan et al. showed that the shamrock approach could prolong the analgesic time after hip surgery [24].

Case reports have suggested that local injection of anesthetics into the quadratus lumborum can effectively relieve pain in patients after various hip and lower limb surgeries [25–28]. As shown in the present study, the dose and requirements of sufentanil in patients administered QLB at different time intervals after surgery were significantly lower than those in the control (L) group. Meanwhile, there were no significant differences between the two groups in sufentanil dose and resting and active state VAS scores at various time points after surgery (all lower than 3/10, *P* > 0.05), suggesting that compared with ultrasound-guided LPB, ultrasound-guided QLB provides similar post-operative analgesia after hip arthroscopy. These findings corroborated a recent placebo-controlled trial demonstrating that ultrasound-guided QL3 block represents an effective pain management method following THA [13]. However, it should be noted that the present study is the first to compare QLB and the widely used LPB with a prospective controlled design.

Although LPB usually has few complications [29], it may be associated with serious adverse events, with epidural spread being the most common complication [30]. In a recent study, 5 of 17 volunteers administered LPB had MRI-confirmed distribution of the drug in the epidural space [31]. In this trial, a 52-year-old female patient in the L group developed epidural spread with stable hemodynamics during surgery and no other discomfort postoperatively. After 6 h of the block, the epidural effect disappeared and the patient reported no specific discomfort, suggesting a good post-operative analgesic effect. However, after 24 h, this individual showed a slightly higher VAS score compared with the remaining patients of the same group. The reason may be that after the anesthetic spread to the epidural area, the drug concentration around the lumbar plexus was reduced, not effectively providing prolonged analgesia. QLB is a typical intramuscular drug injection approach, and its effects can spread through the thoracolumbar fascia to the paravertebral space or directly affect the transverse abdominis level [32]. The needle approach and the location of local anesthesia are not far from the abdominal cavity, internal abdominal organs and large blood vessels, and local anesthetics are not directly injected into the adjacent areas of the large nerve [32]. Therefore, the likelihood of various complications is much lower than that of other nerve blocks. So far, serious complications have not been reported, including in the present study, indicating that QLB might be safe, but a comparison with LPB will have to be made.

An unnecessary femoral nerve block is considered a possible complication of QLB3. A reasonable theoretical explanation lies in the direct anatomical continuity of Thoracolumbar fascia and iliac fascia and the possibility of anesthetic spreading downwards along the iliac fascia, resulting in quadriceps weakness [33–36]. QLB3 without puncturing the psoas major muscle fascia (PMM) was performed, and the contrast agent could not spread to the tail end [27]. This finding indicates that avoiding PMM perforation results in no extra quadriceps weakness. In this trial, muscle strength was 4.0 (4.0–4.0) at 30 min after QLB, demonstrating that it was unaltered. It may be that anesthesiologists in this trial were all experienced in ultrasound-guided nerve block and may not puncture the PMM during the block, so the local anesthetic did not spread to the tail end, thus avoiding quadriceps weakness. As shown above, the muscle strength after surgery in the L group was 2.0 (1.0–3.0), which was significantly lower than that of the Q group (*P* < 0.05). These data suggest that QLB has little to no impact on specific muscle strength, which is a major advantage of this approach over LPB. As a result, the risk of falling after surgery can be reduced, hip surgery patients could get out of bed as early as possible, and complications could be prevented after QLB.

The limitations of this study should be mentioned. First, it only assessed the clinical efficacies of QLB and LPB in hip arthroscopy. Whether QLB and LPB provide similar analgesic effects in other types of surgery needs further investigation. Secondly, although this was a randomized prospective trial, all patients were treated in the same hospital. Thirdly, we did not correct for multiple comparisons during the analysis of secondary endpoints because of the small sample size of this exploratory study. In addition, the study might have been underpowered for the secondary endpoints. Therefore, multicenter randomized prospective trials should be conducted to confirm our findings. Finally, the mechanism of action of QLB remains unclear, and more trials are needed to explore its analgesic mechanism for promoting the clinical application of this approach.

In conclusion, compared with ultrasound-guided LPB, ultrasound-guided QLB provides similar, good post-operative analgesia after hip arthroscopy, with less impact on muscle strength. Complications will have to be examined in future trials. However, further research is needed to explore whether it could replace LPB to provide perioperative analgesia in other hip surgeries.

## Data Availability

The datasets used and/or analyzed during the current study are available from the corresponding author on reasonable request.
